# Case 4/2018 – Important Mitral Valve Regurgitation Caused by Hammock Mitral Valve in 8 Year-Old Girl

**DOI:** 10.5935/abc.20180128

**Published:** 2018-07

**Authors:** Edmar Atik, Alessandra Costa Barreto, Maria Angélica Binotto, Renata de Sá Cassar

**Affiliations:** Instituto do Coração do Hospital das Clínicas da Faculdade de Medicina, Universidade de São Paulo, São Paulo, SP – Brazil

**Keywords:** Heart Defects, Congenital / surgery, Mitral Valve Insufficiency, Heart Murmurs, Echocardiography, Electrocardiography, X-Rays

## Clinical data

Heart murmur was auscultated during routine examination at 6 years of age, with complaints of tachycardia and chest pain at that time. A diagnosis of mitral regurgitation caused by hammock mitral valve was performed and enalapril 2.5 mg/day (0.1 mg/kg) was started. She reported being asymptomatic, capable of performing physical activity.

**Physical examination**: good overall health status, eupneic, acyanotic, normal pulses in the four limbs. Weight: 25 kg; height: 130 cm; right upper limb blood pressure: 90 x 60 mmHg; Heart Rate (HR): 96 bpm; Oxygen Saturation (O_2_Sat): 97%.

**Precordium**: diffuse apex beat, palpated in the sixth left intercostal space, deviated from the midclavicular line and with systolic impulses in the left sternal border. Muffled heart sounds, holosystolic murmur in the mitral and axillary regions with a diastolic rumble after the third heart sound, both of moderate intensity. Liver palpable at the right costal margin, painless.

### Complementary examinations

**Electrocardiogram:** sinus rhythm, with signs of overload of the left cavities. High R waves, preceded by positive Q waves and with normal T waves, in the left leads, indicating left ventricular (LV) diastolic overload. The P wave was negative at V1 and V2 and enlarged at the other leads. Ventricular repolarization was normal. AQRS + 30º; AP + 50º and AP +60º.

**Figure 1:**
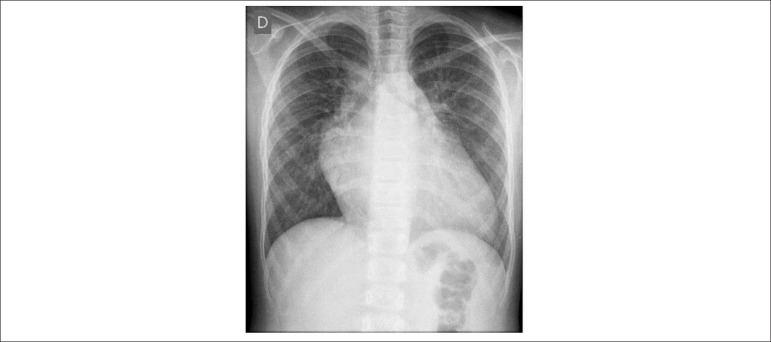
Chest X-ray showing a clear increase in the cardiac area at the expense of the left heart cavities and the prominent pulmonary vascular network in the upper pulmonary area, indicating pulmonary congestion.

**Chest X-ray:** Increase in the cardiac area at the expense of the left heart cavities and with prominent pulmonary vascular network in the upper pulmonary area, indicating of pulmonary venocapillary congestion (Figure 1).

**Figure 2:**
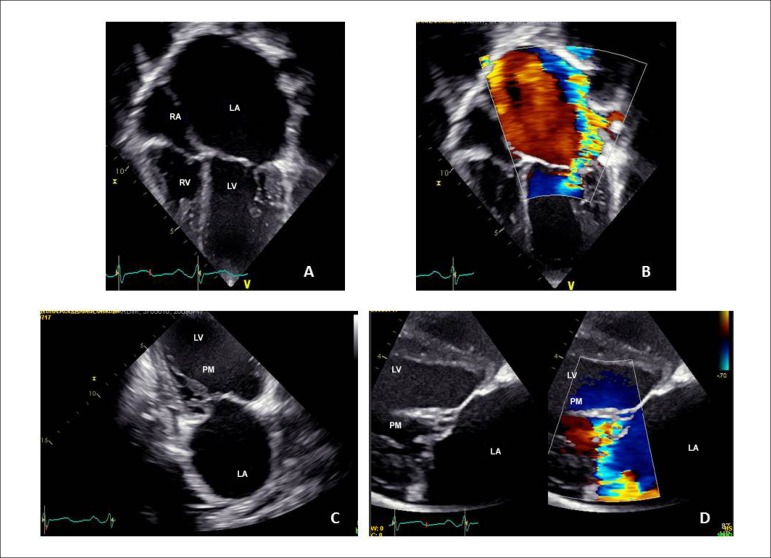
Echocardiogram showing the marked enlargement of the left heart cavities, especially the left atrium in A and B, due to the evident mitral regurgitation in B. The close connection of the leaflets with the papillary muscle occurs without chordae tendineae in C and D. RA: right atrium; LA: left atrium; RV: right ventricle; LV: left ventricle; PM: papillary muscle.

**Echocardiogram:** showed markedly dilated left cavities. The mitral valve was thickened, with short chordae tendineae and with leaflets almost attached to the two papillary muscles (hammock mitral valve). The mitral annulus was thickened, with important valve regurgitation, which allowed the appearance of a maximum diastolic gradient of 28 mmHg and a mean of 10 mmHg. The pulmonary arteries were confluent and slightly dilated (13 mm). MPAP: 36 mmHg; Right Ventricle (RV): 12; LV: 56; Left Atrium (LA): 59; Ao: 17; septum and posterior wall: 7; LV Ejection Fraction (LVEF): 63%; mitral annulus: 30; tricuspid annulus: 21 mm (Figure 2).

**Clinical diagnosis:** important mitral regurgitation caused by hammock mitral valve with enlargement of the left heart cavities in an 8-year-old girl without apparent symptoms.

**Clinical rationale:** There were clinical elements pointing to a diagnosis of important mitral valve regurgitation, related to the presence of a regurgitation systolic murmur and diastolic rumble in the mitral area and in the axillary region. The clinical effect was accentuated due to the large increase of the left heart cavities, disclosed by the usual complementary examinations. The diagnosis was well established by the echocardiography regarding the congenital etiology of the defect in the anatomical characterization of the hammock mitral valve. It was observed that, despite the marked consequence of the defect, the patient remained symptom-free and under natural evolution until 8 years of age.

**Differential diagnosis:** with the diagnostic characterization of marked mitral valve regurgitation, the differential diagnosis refers to the search for its etiology. At this age, one should remember the rheumatic cause, even without suggestive prodromes. Other causes may be related to mitral valve prolapse, valve lesion due to endocarditis or an ischemic lesion of anomalous origin in the left coronary artery directly from the pulmonary trunk.

**Conduct:** Considering the marked consequence of the mitral valve defect, there was a surgical indication aimed to correct the defect and prevent more severe disease evolution alterations, such as ventricular dysfunction, pulmonary artery hypertension and cavity thrombosis with systemic embolism, among the main ones. It was presumed that the most appropriate technique would be the mitral valve replacement, which was markedly affected, but with a chance of success through a plasty procedure, to be evaluated at the time of the surgery.

## Comments

The hammock mitral valve was first described as a direct connection of the papillary muscles with the mitral leaflets, either directly or by the interposition of unusually short chordae tendineae. This congenital malformation of the tensile system is sometimes called a “hammock mitral valve”, as it mimics a hammock when it is observed from the atrium. The chordae tendineae are thick and extremely short, reducing inter-cordial spaces and leading to an abnormal excursion of the leaflets, which can cause stenosis and regurgitation.

When the space between the abnormal chordae is completely obliterated, a fibrous and muscular bridge joins the two papillary muscles. In its most severe form, with no chordae tendineae, the papillary muscles are directly fused with the free margin of the leaflets. Mitral regurgitation progressively worsens, with or without concomitant stenosis. However, even with these anatomical alterations, the valve may show a relatively normal function for many years, as shown by some recent findings.^1^

Most reported cases are in the pediatric age group, and there are only a few reports of hammock mitral valve anomaly in adults. In cases of hammock mitral valve, the repair can be performed through annuloplasty, commissurotomy, modified techniques of posterior annulus shortening and papillary muscle division, according to the presentation of the valve apparatus morphology.^2-4^
